# Comparison of health information systems capacity among China and Association of Southeast Asian Nations countries based on the World Health Organization SCORE assessment tool

**DOI:** 10.7189/jogh.15.04130

**Published:** 2025-06-02

**Authors:** Jing Kang, Chenting Zhu, Wenbing Ouyang, Lingbo Huang, Qiming Feng, Yujun Chen, Qian Huang, Ruizhao Lu, Xianjing Qin, Jun Feng

**Affiliations:** 1 Health and Policy Research Center, Guangxi Medical University, Nanning, Guangxi, China; 2 School of Nursing, Guangxi Medical University, Nanning, Guangxi, China; 3 The First Affiliated Hospital of Guangxi Medical University, Nanning, Guangxi, China; 4 School of Public Health, Guangxi Medical University, Nanning, Guangxi, China; 5 Business school, Hongik University, Jochiwon-eup, Republic of Korea

## Abstract

**Background:**

This study examines the current state of health information system (HIS) capacity in China and 10 Association of Southeast Asian Nations (ASEAN) countries, identifying successful practices and challenges to inform policy recommendations and support health information cooperation between China and ASEAN.

**Methods:**

The HIS capacity between China and ASEAN countries was summarised and compared through a descriptive analysis using the World Health Organization (WHO) Survey, Count, Optimize, Review and Enable (SCORE) technique package. We analysed the universal health coverage (UHC) index, health-related Sustainable Development Goals data availability, and five essential interventions for correlation using Spearman’s correlation coefficient. We obtained the data from the WHO SCORE database and WHO Global Health Observatory.

**Results:**

All countries achieved comprehensive and sustainable levels in data accessibility, indicating well-established data for health-related Sustainable Development Goals and UHC indicators. However, fewer than half of the countries achieved comprehensive or sustainable levels in Count, Optimize, and Enable dimensions. China and Malaysia scored highest across all five dimensions, with China reaching sustainable levels in Optimize and Review, and Malaysia achieving similar in Review. Singapore and Laos exhibited lower scores in Enable and Count, respectively. No country reached sustainable levels across all dimensions, highlighting areas for improvement in HIS capacity across the region. The Count dimension showed a significant positive correlation with the UHC Index.

**Conclusions:**

HIS capacities in Count, Optimize, and Enable are weak across China and ASEAN, suggesting the need for targeted efforts to improve health data collection, analysis, and policy application. Strengthening the link between HIS and national health planning is crucial for advancing HIS development. There is also a need to actively improve the cooperation and sharing mechanisms for health information, and promote the exploration of establishing cooperation agreements and memorandums between China and ASEAN countries to enhance the availability, reliability, and wide dissemination of shared data.

Access to accurate, reliable, and up-to-date health information is essential for individual and public health. It underpins critical areas such as health care management, public health safety, policy-making, quality health care delivery, and health education [1–3]. Evidence indicates that effective health information is pivotal in achieving universal health coverage (UHC) and the health-related Sustainable Development Goals (SDGs), as it enhances health knowledge and promotes positive health behaviours [4,5]. Conversely, limited access to timely and accurate health information significantly contributes to morbidity and mortality, particularly in low- and middle-income countries [6]. The COVID-19 pandemic has further highlighted the importance of health data and information [7,8].

Strong health information systems (HIS) are the backbone of effective health data acquisition and management as well as digital health. Health information systems integrate data, processes, and knowledge management into a cohesive framework, forming a critical socio-technical component of health care delivery [9]. However, HIS have evolved in a fragmented manner due to administrative, economic, legal, and donor influences [10]. With the fast developing of health technology and artificial intelligence, countries have made significant strides in developing their HIS in recent years, though facing distinct challenges and are at varying stages of implementation [7]. However, HIS are still limited worldwide, especially in those developing countries [11,12].

The Association of Southeast Asian Nations (ASEAN), comprising ten Southeast Asian countries, namely Brunei, Cambodia, Indonesia, Laos, Malaysia, Myanmar, Philippines, Singapore, Thailand, and Vietnam, represents a region marked by significant political, economic, and socio-cultural diversity. In recent years, both China and ASEAN countries have prioritised HIS development. China has developed a multi-tiered health information platform spanning national, provincial, municipal, and county levels, and the telemedicine played a crucial role in medical resource allocation during the COVID-19 pandemic [13]. Within ASEAN, HIS development is diverse. High-income countries like Singapore, Malaysia, and Thailand have implemented comprehensive digital health frameworks integrating electronic medical records and telemedicine services. In contrast, countries such as Indonesia, the Philippines, and Vietnam are in the early stages of digitising health records and establishing national HIS [14]. The rapid growth in health care needs, coupled with advancements in digital health systems, big data analytics, and artificial intelligence, presents both opportunities and challenges for ASEAN countries. While significant progress has been made in digital health transformation, gaps remain in achieving fully integrated and effective HIS. All ASEAN member states have laid the groundwork for e-health development and increasingly recognise digital health’s importance in national policy [15,16].

Despite progress, many countries in the region still lack comprehensive HIS capable of consistently collecting, analysing, and utilising high-quality data to monitor health factors and support effective interventions. This study aims to evaluate the current status and capacity of HIS in China and ASEAN countries, identify strengths and gaps, and propose strategies to enhance HIS development, improve health systems, and foster regional cooperation.

## METHODS

### WHO SCORE for health data technical package

Survey, Count, Optimize, Review, and Enable (SCORE) global report 2020 measures the HIS capabilities of 133 countries worldwide by adopting the WHO SCORE for health data technical package for the first time [7]. The World Health Organization SCORE for health data technical package is a comprehensive framework which aims to help countries systematically assess the performance of their HIS, identify gaps, and implement improvements to achieve better health outcomes. The acronym ‘SCORE’ stands for Survey, Count, Optimize, Review, and Enable, which are the five essential interventions of the SCORE for health data technical package. ‘Survey’ assesses population health and risks; ‘Count’ tracks births, deaths, and causes of death; ‘Optimize’ aims to enhance health service data; ‘Review’ evaluates progress and performance; and ‘Enable’ facilitates data use for policy and action, each of the interventions is subdivided into specific elements and can be evaluated by using indicators (**Table 1**) [17]. Among five key interventions, S, C and O prioritise strengthening core data sources and ensuring data availability and quality, whereas R and E focus on advancing the synthesis, analysis, accessibility, and application of health data.

**Table 1:** SCORE interventions and elements [7]*

Intervention		Element
Survey	Survey populations and health risks	S1. System of regular population-based health surveys
		S2. Surveillance of public health threats
		S3. Regular population census
Count	Count births, deaths and causes of death	C1. Full birth and death registration
		C2. Certification and reporting of causes of death
Optimize	Optimise health service data	O1. Routine facility and reporting system with patient monitoring
		O2. Regular system to monitor service availability, quality and effectiveness
		O3.1. Health service resources: health finance
		O3.2. Health service resources: health workforce
Review	Review progress and performance	R1. Regular analytical progress and performance reviews, with equity
		R2. Institutional capacity for analysis and learning
Enable	Enable data use for policy and action	E1. Data and evidence drive policy and planning
		E2. Data access and sharing
		E3. Strong country-led governance of data

SCORE − Survey, Count, Optimize, Review, and Enable

*We obtained the permission from World Health Organization to use the Table.

The scoring framework of the interventions is based on a maturity model, wherein the HIS capacity of a country is evaluated across five core interventions and ultimately assigned a score 1–5 which corresponding to five grades: nascent, limited, moderate, well-developed and sustainable. A score of one indicates nascent health information infrastructure, while a score of five signifies fully institutionalised and sustainable capabilities [17].

### Availability of latest data to monitor the health-related SDGs

The SCORE package also evaluates the availability of latest data for monitoring the health-related SDGs and UHC by using 52 SDG indicators and one UHC tracer variable. Each indicator receives a score of one if data availability has been documented in the country. The total number of indicators is summed and is divided by the maximum possible score (53 indicators, representing all contextually relevant indicators for the country) to calculate a percentage. Data availability is then also classified into five capacity levels: nascent (<25%), limited (25–39%), moderate (40–59%), well-developed (60–79%), and sustainable (≥80%) [17].

### Statistical analyses

Descriptive analysis was conducted to summarise and compare the SCORE capacity between China and ASEAN countries. The scores for each intervention were visualised using radar charts, which provide a comprehensive graphical representation of the strengths and weaknesses across the five dimensions. Meanwhile, we conducted a correlation analysis on the UHC index and the health-related SDGs data availability as well as five essential interventions using the Spearman’s correlation coefficient. The Spearman’s analysis was calculated using IBM SPSS Version 27.0 (IBM Corporation, Armonk, New York, USA, 2020)

### Data source

We extracted data from the 2020 Global Report on Health Data Systems and Capacity and also the country analysis of China and 10 ASEAN member states with data collected between 2013–2018. The universal health coverage index data was sourced from the WHO Global Health Observatory. The UHC index is released biennially, with the latest data from 2021. Since the SCORE data (2013–2018) predates the UHC index, we used the 2019 UHC index for correlation analysis.

## RESULTS

### Results of the overall performance

The development of HIS across China and ASEAN countries demonstrates considerable variability, reflecting differing stages of digital health transformation. Based on the SCORE package, China leads with high performance across all five essential interventions, achieving sustainable levels in Optimize and Review and well-developed levels in Survey, Count, and Enable. Malaysia also exhibits consistent strength, achieving well-developed or sustainable levels across all metrics. Conversely, some ASEAN countries, such as Laos and Myanmar, show moderate development, with significant gaps in Count and Enable, which limit their overall capabilities.

The ASEAN regional average underscores strong progress in Survey, reflecting a focus on health data collection, while Count and Enable highlight critical areas for improvement. Notably, even advanced economies like Singapore reveal disparities, with a low score in Enable despite higher ratings in Count and Survey. This heterogeneity points to a pressing need for tailored interventions and enhanced regional cooperation to address specific weaknesses, promote interoperability, and strengthen overall HIS capacity to meet complex health demands (**Figure 1**).

### Results of the detailed elements

By comparing the evaluation results of detailed elements among China and ASEAN countries, the strengths and weaknesses of their HIS become evident (**Figure 2**).

#### Survey dimension

China, Malaysia, Indonesia and Myanmar score high across all elements in survey dimension, reflecting strong population-based health survey systems, public health threat surveillance, and population census mechanisms. Singapore and Brunei show moderate performance, possibly due to challenges in comprehensive population-based health monitoring.

#### Count dimension

China, Singapore, Brunei, Malaysia, and Philippine perform well in population registration and statistics. Indonesia, Vietnam, Laos, Cambodia and Myanmar score lower, indicating weaker Civil Registration and Vital Statistics (CRVS) systems, which can lead to data gaps in birth and death registration.

#### Optimisation dimension

China, Malaysia, and Thailand have shown outstanding performance in routine facility and community reporting systems as well as service monitoring systems. Health financing data availability is strongest in Thailand and Philippine, where standardised data on public and private health expenditures and catastrophic expenditures are well-documented. Nearly all countries score high in updating and recording data on health workforce density and distribution. Vietnam, Laos, and Cambodia score lower, pointing to gaps in routine health facility reporting systems and limited health financing data standardisation.

#### Review dimension

China and Malaysia reach the highest sustainable level across all indicators, demonstrating strong analytical and evaluative capacities. Thailand, Indonesia, Vietnam, the Philippines, and Cambodia also perform well. In contrast, Singapore, Brunei, Laos, and Myanmar remained at limited or intermediate levels.

#### Enable dimension

China and Malaysia demonstrate strong capacity in data-driven national health planning, conducting comprehensive reviews of past performance, disease burden, and health system strengths. However, significant improvements are needed in Singapore, Brunei, Cambodia, and Myanmar in utilising data to enhance health policy, as well as in the accessibility and sharing of health data.

### Results of the availability of latest data to monitor the health-related SDGs

The availability of health data also varies significantly across China and ASEAN countries. Malaysia and Cambodia demonstrate the highest levels of data completeness, while the Philippines, Myanmar, and Laos exhibit moderate availability, reflecting progress in data collection efforts alongside persistent gaps. Conversely, China, Singapore, Indonesia, Vietnam, Thailand, and Brunei demonstrate relatively low data availability (**Table 2**).

**Table 2:** Results of the health-related SDGs data availability among China and ASEAN countries

World Bank country income levels	Country	Number of accessible indicators	Total relevant indicators	Proportion of accessible indicators (%)	Capability maturity rating	Capability maturity evaluation
Upper-middle income	China	34	53	64	4	Well-developed
High-income	Singapore	34	53	64	4	Well-developed
	Brunei	40	51	78	4	Well-developed
Upper-middle income	Malaysia	51	52	98	5	Sustainable
	Thailand	43	52	83	5	Sustainable
	Indonesia	36	53	68	4	Well-developed
Lower-middle income	Vietnam	41	53	77	4	Well-developed
	Philippines	42	53	79	4	Well-developed
	Laos	41	53	77	4	Well-developed
	Cambodia	46	53	87	5	Sustainable
	Myanmar	37	51	73	4	Well-developed

ASEAN – Association of Southeast Asian nations, SDG − Sustainable Development Goals

Regarding key indicators, communicable disease data are generally well reported, but HIV/AIDS, neglected tropical diseases (NTDs), and hepatitis B coverage have inconsistencies. Non-communicable diseases are underreported in most ASEAN countries, except for Brunei and Malaysia. Health workforce data are particularly weak in China, Myanmar, Laos, and Cambodia. Environmental health risks, air pollution, and water quality data are incomplete for many countries. Government spending and financial protection data are among the most inconsistent indicators.

### Spearman’s rank correlation analysis between UHC Index and the SCORE capacity

The Spearman’s correlation analysis reveals that, among the relationships between the UHC Index and SCORE capacity, only Count shows a significant positive correlation with the UHC Index. The correlation coefficient is 0.693, with a *P*-value of 0.018, suggesting that improved Count capabilities can positively influence the implementation of UHC and support policy development (**Table 3**). However, other interventions of Survey, Optimize, Review and Enable show weak or insignificant correlations with the UHC Index.

**Table 3:** Correlation results between UHC Index and the SCORE capacity

Variables	Correlation coefficient	*P*-value
Health-related SDGs data availability	−0.096	0.779
Survey	−0.259	0.442
Count	0.693	0.018
Optimize	0.156	0.648
Review	0.092	0.788
Enable	−0.086	0.802

SCORE − Survey, Count, Optimize, Review, and Enable, SDG − Sustainable Development Goals, UHC − universal health coverage

The small sample size (only 11 samples) likely contributes to the weak or insignificant correlations observed, as smaller samples are more prone to random factors and have lower statistical power. Expanding the sample size and collecting more data would improve the accuracy of future evaluations.

## DISCUSSION

### HIS in China and ASEAN: progress and shared challenges

China and ASEAN countries have recognised the critical importance of HIS and began strengthening their frameworks in the late 20th century. These systems now incorporate national health data, public health information, medical service records, and health system resources. High- and upper-middle-income countries like Singapore, Malaysia, and Thailand have developed more advanced systems due to earlier initiatives. Singapore’s Synapxe platform, for instance, facilitates seamless data sharing among government bodies, health care institutions, and patients, proving invaluable during the COVID-19 pandemic [18]. Similarly, Brunei has made notable advancements with its integrated HIS and a strong focus on telemedicine [19]. However, lower-middle-income countries, such as Cambodia, face persistent challenges, including inadequate infrastructure and limited capacity. These issues contribute to fragmented health information, leading to inefficient resource allocation, delayed detection of communicable diseases, and constrained efforts to integrate UHC and Global Health Security initiatives [20,21].

The WHO SCORE assessment of HIS in China and ASEAN further highlights the need for improvement. While HIS development seeks to support policy-making and enhance health care quality, over half of the countries in the region exhibit limited or moderate capacities in data utilisation. Persistent challenges, including fragmented data collection, weak governance and data-sharing frameworks, and poor data timeliness, continue to hinder progress [22,23]. Addressing these common barriers will be crucial for strengthening HIS and achieving more efficient, resilient health care systems across the region.

### Great variation in HIS development across countries

China leads in all five essential interventions, likely due to its centralised public health system and significant investment in digital health [24]. Malaysia performs consistently well among ASEAN countries, benefiting from a strong regulatory framework and effective cross-sector collaboration. Less developed countries like Laos and Myanmar face major gaps in Count and Enable, reflecting weak infrastructure, limited funding, and a fragmented health system.

Notably, upper-middle and lower-middle-income countries such as Malaysia, Indonesia, Cambodia and Myanmar performed better than high-income nations like Singapore and Brunei in the WHO SCORE evaluation. While Singapore boasts an advanced HIS infrastructure with abundant and timely data [25], its performance in critical areas such as population health surveys, service monitoring, and data utilisation remains limited. Singapore’s lower-than-expected performance highlights the complexity of the SCORE framework’s orientation and structural differences in national health systems as well as different health information priorities. For example, the ‘Enable’ intervention emphasises data-driven policy support, including cross-sector integration, transparency, and systematic application in national health planning. Despite Singapore’s high digitalisation, its health data usage prioritises clinical service optimisation over public policy design [26,27]. Meanwhile, Singapore’s advanced health care system with significant private sector involvement complicates health data management. Weak public-sector coordination and reliance on voluntary private-sector participation limit sharing of health data [28]. What’s more, for those lower-middle income countries like Cambodia and Myanmar, they have received substantial international assistance to strengthen their HIS, including CRVS, surveillance systems and also national health surveys.

These findings underscore the uneven progress in building robust HIS and the need for tailored approaches to address country-specific challenges.

### Key focus areas for HIS capacity improvement

Analysis of the five essential interventions highlights the particular need for China and ASEAN countries to strengthen the Count and Enable capacity. In the Count dimension, a robust basic registration system is crucial for the foundation of health care planning. The high registration rates in Malaysia and Thailand are directly linked to their well-established CRVS systems. In contrast, the shortcomings in Laos and Myanmar may lead to biased estimates of health needs and misallocation of policy resources. Low adoption of death medical certificates and International Classification of Diseases (ICD) codes further weaken disease burden analysis. Hence, countries should prioritise CRVS system development [29], seek international support, and enhance death certification training in medical education.

For the Enable dimension, data-driven decision-making remains a bottleneck. While most countries have health data portals, coverage and accessibility are often insufficient which limits their impact on policy. To address this, countries should promote ‘data as a public good,’ establish mandatory openness standards, and enhance cross-departmental coordination [30].

### Strengthen commitments to HIS development

Strong HIS are essential for tracking national health priorities, ensuring health security, and providing equitable access to quality health care. The Spearman’s analysis shows a significant positive correlation between Count capacity and the achieving of UHC, confirming the critical role of foundational data systems in achieving UHC. Recognising this, the 58th World Health Assembly in 2005 emphasised the need to develop health information and communication technology infrastructure [31]. The United Nations 2030 Agenda for Sustainable Development further highlights the transformative potential of information technologies in accelerating progress, bridging digital divides, and fostering knowledge societies [32]. Similarly, the WHO Global Strategy for Digital Health (2020–2025) underscores the importance of accessible, scalable, and sustainable digital health solutions to address pandemics, strengthen health infrastructure, and advance health care access, aligning with health-related SDGs [33].

To realise these goals, governments in China and ASEAN must intensify their commitment to HIS development by mobilising resources, establishing clear objectives, and implementing actionable frameworks. This includes creating a supportive environment for health innovation and ensuring strong government leadership to drive health system improvements. Lower-middle-income countries such as Laos, Cambodia, and Myanmar should prioritise their role as primary drivers and regulators of HIS, focusing on legal frameworks, strategic planning, and data security to build resilient systems. Moreover, low- and middle-income countries can benefit from establishing a checklist of contextual factors to guide the development of digital health initiatives and HIS capacity-building efforts [34]. Across all nations, particular attention should be given to addressing weaker HIS dimensions-Count, Optimize, and Enable-by utilising SCORE assessments to identify and close gaps. Strengthened commitments to these areas will not only enhance health information capacity but also ensure that HIS can effectively support health priorities and improve outcomes across the region.

### Promote China-ASEAN health information cooperation: overcoming challenges for enhanced data sharing

Robust data sharing mechanisms are crucial for informed decision-making and coordinated responses to public health challenges and regional health security. The COVID-19 pandemic has underscored the essential role of timely and accurate health information in global health governance. It has emphasised the need for better access to and sharing of health data, not only between countries but also across various sectors within nations.

Despite its importance, regional cooperation in health information faces significant obstacles. National security concerns, political differences, and shifting global power dynamics, particularly regarding ASEAN’s position, complicate collaboration. Additionally, disparities in HIS and data collection capacities, combined with cultural and language differences, further hinder effective cooperation.

To overcome these challenges, China and ASEAN must prioritise building mutual trust and fostering open communication. Strengthening mechanisms for health information collaboration and establishing clear agreements or memorandums of understanding will provide a foundation for effective partnerships. These initiatives can improve the availability, reliability, and dissemination of shared health data, paving the way for stronger regional HIS and more unified responses to future health threats [35].

### Limitations

There are several limitations for this study. First, the study relies on data from 2013–2018, and as a result, it does not capture the transformations in HIS that have occurred due to the COVID-19 pandemic, which has significantly impacted health systems globally. Second, constrained by data gaps and the complexity of the SCORE framework, the study primarily adopts a descriptive analysis, which provides a broad overview of HIS but limits the depth of insights. While, WHO has been constantly updating the SCORE technical package. Looking ahead, future research could address these shortcomings by utilising advanced statistical techniques or machine learning models for deeper insights. Additionally, collaborating with national and regional health authorities for more comprehensive data could help close existing gaps and improve the robustness of the evaluation framework.

## CONCLUSIONS

This study analysed the status quo of the construction of HIS in China and ASEAN countries. China and ASEAN countries have established foundational HIS frameworks but still face challenges in achieving sustainability across all SCORE dimensions. Middle-income countries outperform high-income nations in some aspects, suggesting potential discrepancies in evaluation processes. However, significant gaps in data collection, optimisation, and empowerment remain across the region. Strengthened commitments, targeted interventions, and collaborative efforts are essential to building sustainable and equitable HIS that support health security and policy effectiveness in the region.

**Figure 1.** The Score of Five Intervention of health information system (HIS) among China and Association of Southeast Asian nations (ASEAN) member states.

**Figure 2.** Results of Survey, Count, Optimize, Review and Enable (SCORE) assessment among China and Association of Southeast Asian nations (ASEAN) countries.
